# Supplementary material for: Rapid Biodistribution of Fluorescent Outer-Membrane Vesicles from the Intestine to Distant Organs via the Blood in Mice
Source: Int J Mol Sci. 2024 Feb 2;25(3):1821. doi: 10.3390/ijms25031821 (PMC10855177; doi:10.3390/ijms25031821)
Supplement: Supplementary file 1 [file ijms-25-01821-s001.zip › ijms-2784424-supplementary.pdf]

Supplementary materials

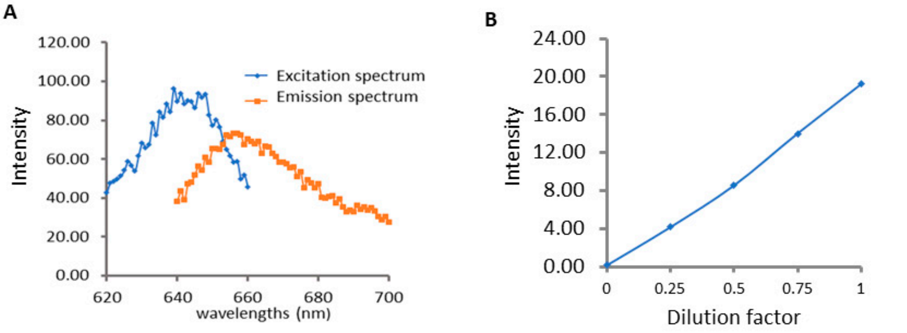

Figure S1: miRFP713-OMV spectra (A) and linearity of miRFP713-OMV fluorescence (B).

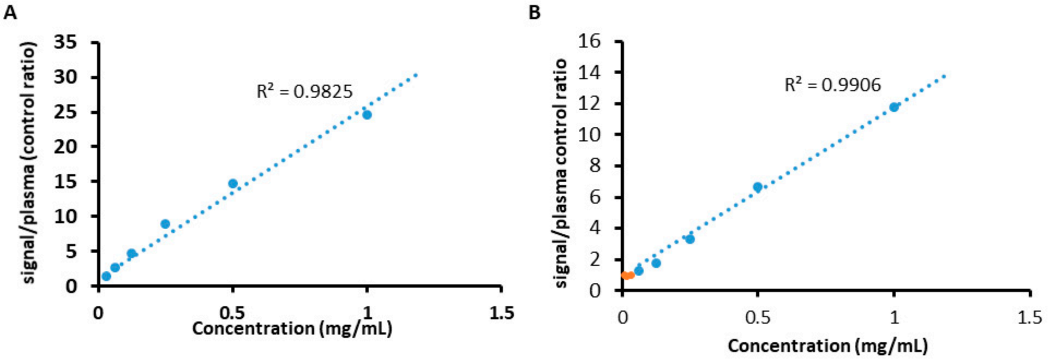

Figure S2: Calibration of the LUMINA III (PerkinElmer) in vivo imaging platform before tracking the fluorescence emitted by BALB/C mice injected (A) versus gavaged (B) with miRFP713-OMVs. A. In vitro detection limit of miRFP713-OMV (used for i.v. studies) signals to noise ratios detected on the fluorescence device LUMINA III (PerkinElmer) ex: 620 ± 10 nm; em: 670 ± 20 nm. Preparation of a wide range of concentrations of the compound by serial dilutions to evaluate the fluorescence intensity of miRFP713-OMVs. The linearity of the fluorescence detection and the detection limit were evaluated using 10 µL samples: Pure, 1/2, 1/4, 1/8, 1/16, 1/32, 1/64, 1/128, 1/256 (dilutions in murine plasma). The minimal detection was reached for a concentration of 0.063 mg/mL (dilution 1/16) (miRFP713-OMV signal/background ratio = 2.73). B. In vitro detection limit of miRFP713-OMV (used for per os studies) signals to noise ratios detected on the fluorescence device LUMINA III (PerkinElmer) ex: 620 ± 10 nm; em: 670 ± 20 nm. A wide range of concentrations of the compound by serial dilutions to evaluate the fluorescence intensity of miRFP713-OMVs, the linearity of the fluorescence detection and the detection limit were evaluated using 10 µL samples: Pure, 1/2, 1/4, 1/8, 1/16, 1/32, 1/64, 1/128, 1/256 (dilutions in murine plasma). The in vitro detection limit of miRFP713-OMV using the LUMINA III (PerkinElmer) platform at ex: 620 ± 10 nm / em : 670 ± 20 nm was reached for a concentration of 0.063 mg/mL (dilution 1/16) (miRFP713-OMV signal/background ratio = 1.25).

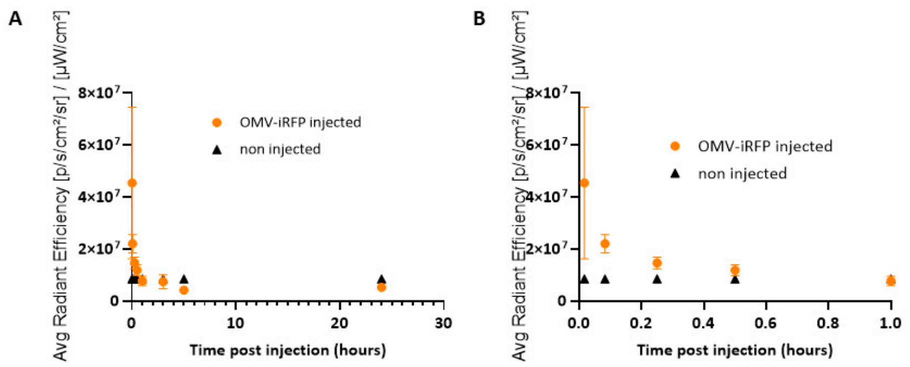

Figure S3: Fluorescence signals in plasma aliquots recovered from blood sampled 20 h (**A**) or 1 h (**B**) after intravenous injection of miRFP713-OMV in healthy mice. The Diffusion and elimination half-life is 1.16 min when the pure elimination half-life is 11.78 min (using Graphpad Prism 9.3).
